# Passive Smoking and Risk of Gestational Diabetes Mellitus among Nonsmoking Women: A Prospective Cohort Study in China

**DOI:** 10.3390/ijerph19084712

**Published:** 2022-04-13

**Authors:** Jigen Na, Huiting Chen, Hang An, Mengyuan Ren, Xiaoqian Jia, Bin Wang, Zhiwen Li, Xiaohong Liu, Rongwei Ye, Nan Li

**Affiliations:** 1 Key Laboratory of Reproductive Health, National Health Commission of the People’s Republic of China, Institute of Reproductive and Child Health, Peking University, Beijing 100191, China; najigen@bjmu.edu.cn (J.N.); htchen1999@163.com (H.C.); anhang@bjmu.edu.cn (H.A.); myren@bjmu.edu.cn (M.R.); jiaxq.only@foxmail.com (X.J.); binwangpku@foxmail.com (B.W.); yerw@bjmu.edu.cn (R.Y.); linan01@pku.edu.cn (N.L.); 2 Department of Epidemiology and Biostatistics, School of Public Health, Peking University, Beijing 100191, China; 3 Beijing Haidian Maternal and Child Health Hospital, Beijing 100080, China

**Keywords:** passive smoking, gestational diabetes mellitus, pregnant women, parity, cohort study

## Abstract

Background: Increasing evidence has shown that active smoking can increase the risk of gestational diabetes mellitus (GDM), but the effect of passive smoking is still unknown. Women in pregnancy are vulnerable to secondhand smoke. This study explored the association of passive smoking with GDM in China. Method: A total of 3083 nonsmoking pregnant women living in Beijing were recruited into a prospective cohort study. Sociodemographic and passive smoking data were collected with structured questionnaires during face-to-face interviews. Glucose levels were measured by physicians according to standard protocols. Multivariate logistic regression was performed for the association estimation after accounting for potential confounders. Result: In total, 562 of the 3083 participants developed GDM (18.23%); 779 participants (25.27%) reported exposure to passive smoking. After adjusting for age, BMI, ethnicity, education, occupation, and parity, passive smoking conferred an approximately 1.4-fold risk increase in GDM (adjusted odds ratio (OR) = 1.37, 95% confidence interval (CI): (1.11, 1.70)). The adjusted ORs with 95% CIs for passive smoking levels of <1, 1–6, and ≥7 times per week were 1.21 (0.94, 1.55), 1.81 (1.22, 2.69), and 1.70 (1.02, 2.84), respectively. An obvious passive-smoking–GDM association was observed among only nulliparous women (adjusted OR = 1.45, 95% CI: (1.14, 1.85)). Conclusion: Frequent exposure to secondhand smoke could increase the risk of GDM among nonsmoking pregnant women. Parity status might modify their association. Public policies should be advocated to prevent passive smoking among this population.

## 1. Introduction

Gestational diabetes mellitus (GDM) refers to glucose intolerance with onset or first recognition during pregnancy [1]. The prevalence of GDM has increased in recent years, especially in low- and middle-income countries [2,3], and the current prevalence is approximately 14.7% (by the IADPSG criteria) around the world [4]. It has been reported that nearly twenty-two of one hundred pregnant women have been diagnosed with GDM since the implementation of the universal two-child policy in northern China [5]. GDM not only causes adverse pregnancy outcomes [6,7] but also leads to offspring type 2 diabetes in the short or long term [8]. Finding a solution to this threat is a priority for Millennium Development Goal 5 to improve maternal health by the World Health Organization [9]. As GDM continues to emerge as a major public health concern, interventions during pregnancy would provide important opportunities to improve the health status of mothers and children.

Among the most likely modifiable factors, tobacco smoke is an environmental exposure that is much easier to eliminate. It has been considered that tobacco smoke exposure can impair glucose homeostasis [10,11,12]. Although active smoking is a well-documented risk factor for GDM [12,13,14], evidence for maternal passive smoking has been inconclusive [15,16,17,18]. Some studies suggested that maternal passive smoking could increase the risk of GDM [15,17], whereas others found no obvious association [16,18]. The certain cross-sectional or case–control studies mentioned above are typically inadequate for determining temporal relationships about their association; therefore, it is essential to conduct a well-characterized prospective cohort study to investigate their causal relationship to provide more evidence for tobacco control.

According to a national survey in China, only approximately 3% of women are active smokers, and nonsmoking women are commonly exposed to smoke from men [19]. This fact allows us to study the association of passive smoking with reproductive health in nonsmoking women. In this study, we used data from a prospective cohort study in Beijing, China, to investigate whether passive smoking during pregnancy is associated with GDM. Considering the potential influence of childbearing history on GDM [20], we also assessed whether the association of maternal passive smoking varied on parity.

## 2. Materials and Methods

### 2.1. Study Design and Participants

**Figure 1:**
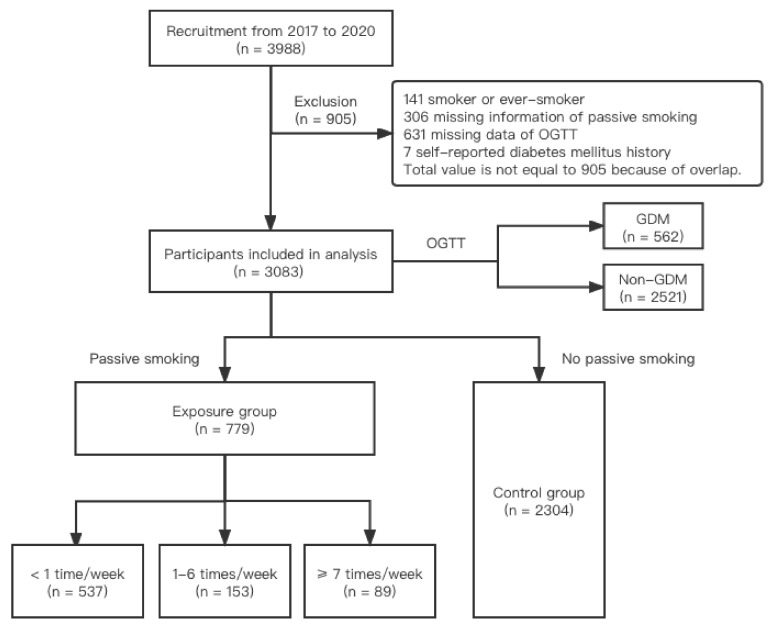
Flowchart of recruitment and exclusion.

This study was conducted in Haidian Maternal and Child Health Hospital in Beijing, China. This is an ongoing prospective cohort study designed to assess the relationship between environmental factors during early pregnancy and preterm birth, as well as other adverse pregnancy outcomes. Detailed information on the cohort was shown in our previous study [21]. Briefly, when a woman came to the hospital for her routine prenatal examination, she was invited if the following criteria were met: (1) aged at least 18 years; (2) enrolled within 20 gestational weeks; (3) registered and planning to deliver in this hospital; and (4) consented to participation. Pregnant women’s demographic and obstetric characteristics and lifestyle information (passive smoking status during pregnancy) were collected during the first registry by trained healthcare professionals who were trained specifically to follow the protocol for this study. We collected relevant information using a structured questionnaire during the face-to-face interview before their 20th gestational week. During the study period, blood glucose was measured by experienced clinicians who were trained specifically for the variable measure in the research. We also collected blood and other biological samples, such as hair and urine, to identify factors associated with GDM. Much of the study was usual care for the women who came to the hospital for their pregnancy, collecting usual clinical information in a standardized fashion to facilitate analysis. A total of 3988 pregnant women were recruited from 2017 to 2020. Of the whole population, we excluded 141 (3.54%) current smokers or ever-smokers; 306 (7.67%) with missing information on passive smoking; 631 (15.82%) with unknown blood glucose information; and 7 (0.18%) women with a self-reported diabetes mellitus history according to the records in the questionnaire. After these exclusions, 3083 targeted participants (77.31% of the enrolled population) were included in the final analysis. Figure 1 shows the process of recruitment and exclusion.

This study was approved by the Biomedical Institutional Review Board of Peking University (IRB00001052-17028). All participants read and signed the Informed and Consent Form.

### 2.2. Definition of Major Variable

Passive smoking in the study was defined as a status in which pregnant women were exposed to secondhand smoke at least one time per week and the duration was at least 15 min per time. We additionally identified three subgroups of the exposure group according to passive smoking frequencies: (1) less than 1 time per week, (2) 1–6 times per week, and (3) no less than 7 times per week.

In this cohort, pregnant women were routinely screened for GDM during 24–28 weeks of pregnancy with a consensus diabetes screening test by regular hospital personnel. They were diagnosed with or without GDM on the basis of the criteria recommended by the International Association of Diabetes and Pregnancy Study Groups (IADPSG) [22]. Briefly, pregnant women underwent a 75 g oral glucose tolerance test (OGTT) during 24–28 gestational weeks in their prenatal examination. The OGTT was performed in the morning after an overnight fast of at least 8 h. GDM was concluded among pregnant women with at least one of the following criteria: fasting blood glucose ≥ 5.1 mmol/L, 1 h blood glucose ≥ 10 mmol/L, or 2 h blood glucose ≥ 8.5 mmol/L.

### 2.3. Statistical Analysis

We compared mean age and BMI and the distribution of parity, ethnicity, education, and occupation levels between the GDM group and the non-GDM group. The characteristics of pregnant women in the different study groups were compared using Student’s *t* test for quantitative variables and the χ^2^ test for categorical variables. We performed logistic regression models to analyze the association between passive smoking and GDM, adjusted for age, BMI, ethnicity, education, occupation, and parity. We also conducted a trend test to verify whether there was a linear increasing tendency in the prevalence of GDM among different subgroups of passive smoking frequency in logistic regression models. As with an independent risk factor for GDM [20], we stratified the participants by parity to further observe the association between passive smoking and GDM in nulliparous and multiparous women. All analyses were conducted with R software (version 4.0.2; R Development Core Team). A two-tailed *p* value < 0.05 was considered significant.

## 3. Results

A total of 3083 pregnant women were included in the final analysis. Of these participants, 779 (25.27%) reported previous exposure to passive smoking, and 562 (18.23%) were diagnosed with GDM. Table 1 listed the basic socioeconomic and obstetric characteristics of the participants. The pregnant women with GDM tended to be older and more obese, and had a higher proportion of the multiparous than those without GDM. However, there was no significant difference between the two groups regarding Han ethnicity, education, and occupation.

Table 2 presented the association between passive smoking and GDM. We found that the pregnant women exposed to passive smoking had a significantly higher GDM rate than those in the non-exposure group (171/779 vs. 391/2304, *p* = 0.002). The crude OR of GDM for passive smokers was 1.38 (95% CI: (1.12, 1.68)). After adjusting for age, BMI, education, occupation, ethnicity, and parity status, the OR was 1.37 (1.11, 1.70). A positive dose–response relationship was observed between exposure frequency and risk of GDM (less than one time per week: OR = 1.21 (0.94, 1.55); one to six times per week: OR = 1.81 (1.22, 2.69); more than seven times per week: OR = 1.70 (1.02, 2.84)) compared with the reference category (no passive smoking). Furthermore, the linear-by-linear association indicated a significant trend (*p* < 0.001).

After adjusting for age, BMI, ethnicity, education, and occupation, the logistic regression model stratified by parity revealed that passive smoking was a significant GDM risk factor for participants with nulliparous status (OR = 1.45, 95% CI: (1.14, 1.85)) but not for those with multiparous status (OR = 1.14, 95% CI: (0.73, 1.78)) (Table 3).

## 4. Discussion

This prospective cohort study examined the association between passive smoking and GDM in a sample of nonsmoking women in Beijing, China. Independent of age, BMI, ethnicity, education, occupation, and parity, passive smoking intensively contributed to GDM risk in a dose-dependent manner. However, no significant group differences appeared in the GDM risk associated with passive smoking less than once per week. The logistic regression models stratified by parity indicated that passive smoking influenced GDM in nulliparous women rather than in multiparous women, which suggested that passive smoking might have an interaction with parity status on GDM.

Tobacco use has brought numerous acute public health problems to pregnant women around the world. A pooled meta-analysis study claimed that prenatal smoking was a risk factor for GDM, of which the OR, with its 95% CI, was 1.38 (1.19, 1.61) [12]. China is a country with high tobacco consumption. The exposure rate of secondhand smoke among pregnant women in China ranged from 38.9% to 75.1% [23], which needs to be taken seriously as a public health problem.

Few previous studies have explored the effects of passive smoking on GDM, and the results are mixed. A cross-sectional study did not observe an obvious association between passive smoking and GDM in China [16], similar to another cohort study in the USA [18]. Nevertheless, Carroll et al. found that passive smoking in both residences and workplaces increased the risk of GDM in a case–control study of northern China [17]. A Chinese cohort study by Leng et al. displayed a temporal causal effect of secondhand smoke with GDM, and they also mentioned that obesity had a synergistic effect with passive smoking on GDM [15]. Our study not only found similar results to those two studies, but also showed a dose–response pattern. Moreover, the intensity of passive smoking on GDM was almost the same as the pooled relative risk of active smoking from a recent meta-analysis [12]. These results revealed to us that passive smoking could bring detrimental health effects and is perhaps more harmful than active smoking in China.

Childbearing history has been found to be a potential hazard factor for GDM [20]; however, no conclusions were made regarding the interaction of parity among the GDM population with passive smoking. We found GDM was associated with passive smoking among nulliparous women, but not significantly among multiparous women, probably due to relatively small samples; however, the adjusted OR among nulliparous women (1.45) was approximately 30% higher than that among multiparous women (1.14), which might still indicate a higher hazard of passive smoking on GDM among women without childbearing history. Therefore, we suppose that for the multiparous women with GDM, parity may play a more important role and masks the association of passive smoking. To our knowledge, the results of our study could reveal that the association of passive smoking on GDM might be modified by parity for the first time.

Several possible mechanisms may explain how passive smoking leads to GDM. Active and passive smoking might confer GDM risk according to similar mechanisms, which nonetheless remain subject to debate. Tobacco smoke can bring about an inflammatory and oxidative stress environment through the increased adherence of macrophages [24,25,26]. Then, proinflammatory cytokines (IL-1β, TNF-α, and so on), and the oxidative stress response activated insulin resistance [27]. Smoking can also negatively influence the function of β-cells with dose-dependent impairment [11]. Constant exposure to passive smoking may also change glucose homeostasis directly due to the malfunction of β-cells [10].

Our study has two major limitations. Nearly all women in this study population were of Han ethnicity and from urban areas in Beijing; therefore, our conclusion should be extrapolated with caution. In addition, maternal exposure to passive smoking was obtained from self-reported measures with no biomarker data (e.g., nicotine or cotinine); as a consequence, possible recall and reporting biases were unavoidable, which might affect the accuracy of our results.

This study also had several strengths. First, this was a prospective cohort study so that it could explain the temporal causal reference to some certain extent. Second, we included only nonsmoking women, allowing us to distinguish the influence of passive versus active smoking. Third, detailed data on passive smoking, as well as clinical records, allowed us to examine associations among different frequencies of passive smoking, as well as parity status, with GDM. We additionally subdivided the exposure into four levels to measure the dose–response relationship, which is distinguished from previous similar studies. Fourth, we excluded women with a history of diabetes mellitus in the final analysis, thus enabling us to prospectively conclude that most of the increased risk of abnormal glucose was due to passive smoking during early pregnancy.

## 5. Conclusions

We demonstrated herein a significant association between maternal exposure to passive smoking more than one time per week and GDM among nulliparous pregnant women. We deem that passive smoking represents an urgent problem for public health in China, particularly with respect to secondhand smoke exposure to women in pregnancy, such that the Chinese government should take measures to increase awareness of the maternal health risks associated with passive smoking.

## Figures and Tables

**Table 1:** The characteristics of pregnant women by blood glucose in China.

Characteristics	Total	GDM	Non-GDM	*p*
	(*n* = 3083)	(*n* = 562)	(*n* = 2521)	
	No.	No. (%)	No. (%)	
Age (years) ^a^	30.69 ± 3.73	31.68 ± 4.12	30.47 ± 3.60	<0.001
BMI (kg/m^2^) ^a^	21.68 ± 3.05	22.60 ± 3.23	21.47 ± 2.97	<0.001
Ethnicity				
Han	2892	527 (20.33)	2365 (81.67)	1.000
Others	189	34 (17.99)	155 (82.01)	
Education				
Master’s or above	877	143 (16.31)	734 (83.69)	0.216
College	1424	273 (19.17)	1151 (80.83)	
High school or below	778	144 (18.51)	634 (81.49)	
Occupation				
Famer/industry/business/service	742	137 (18.46)	605 (81.54)	0.935
Civil servant	1665	298 (17.90)	1367 (82.10)	
Others	640	114 (17.81)	526 (82.19)	
Parity				
Nulliparous	2501	422 (16.87)	2079 (83.13)	<0.001
Multiparous	582	140 (24.05)	442 (75.95)	

Abbreviations: GDM—gestational diabetes mellitus; BMI—body mass index before pregnancy. ^a^ Mean ± standard deviation of continuous variables is shown.

**Table 2:** Passive smoking and incidence rate of GDM.

	Total No.	GDM	Non-GDM	Crude OR (95% CI)	Adjusted OR ^a^ (95% CI)
		No. (%)	No. (%)		
No passive smoking	2304	391 (16.97)	1913 (83.03)	1	1
Passive smoking ^b^ (times/week)	779	171 (21.95)	608 (78.05)	1.38 (1.12, 1.68)	1.37 (1.11, 1.70)
<1	537	106 (19.74)	431 (80.26)	1.20 (0.95, 1.53)	1.21 (0.94, 1.55)
1–6	153	43 (28.10)	110 (71.90)	1.91 (1.32, 2.77)	1.81 (1.22, 2.69)
≥7 ^c^	89	22 (24.72)	67 (75.28)	1.61 (0.98, 2.63)	1.70 (1.02, 2.84)

Abbreviations: OR—odds ratio; CI—confidence interval. ^a^ Adjusted for age, BMI, ethnicity, education, occupation, and parity. ^b^ Exposure to secondhand smoke for at least 15 min per occasion. ^c^ Test for trend (*p* < 0.001): comparison of no passive smoking and passive smoking frequencies of < 1, 1–6, and ≥ 7 times/week between GDM and non-GDM pregnant women.

**Table 3:** Association between passive smoking and GDM stratified by parity.

Parity	GDM	Non-GDM	Crude OR (95% CI)	Adjusted OR ^a^ (95% CI)
	No. (%)	No. (%)		
Total	562 (18.23)	2521 (81.77)	1.38 (1.12, 1.68)	1.37 (1.11, 1.70)
Nulliparous	422 (16.87)	2079 (83.13)	1.43 (1.14, 1.80)	1.45 (1.14, 1.85)
Multiparous	140(24.05)	442 (75.85)	1.16 (0.77, 1.77)	1.14 (0.73, 1.78)

^a^ Adjusted for age, BMI, ethnicity, education, and occupation.

## Data Availability

The data are available in the main text or can be obtained by contacting the corresponding author (Z.L.).
